# Distribution of Fatal Vibrio Vulnificus Necrotizing Skin and Soft-Tissue Infections: A Systematic Review and Meta-Analysis

**DOI:** 10.1097/MD.0000000000002627

**Published:** 2016-02-08

**Authors:** Kuo-Chin Huang, Hsu-Huei Weng, Tien-Yu Yang, Te-Sheng Chang, Tsan-Wen Huang, Mel S. Lee

**Affiliations:** From the College of Medicine, Chang Gung University, Taoyuan (K-CH, H-HW, T-SC, T-WH, MSL); Department of Orthopaedic Surgery (K-CH, T-YY, T-WH); Department of Diagnostic Radiology (H-HW); Department of Gastroenterology, Chang Gung Memorial Hospital, Chaiyi (T-SC); and Department of Orthopaedic Surgery, Chang Gung Memorial Hospital, Kaohsiung, Taiwan (MSL).

## Abstract

*Vibrio vulnificus* necrotizing skin and soft tissue infections (VNSSTIs), which have increased significantly over the past few decades, are still highly lethal and disabling diseases despite advancing antibiotic and infection control practices. We, therefore, examined the spatiotemporal distribution of worldwide reported episodes and associated mortality rates of VNSSTIs between 1966 and 2014. The PubMed and Cochrane Library databases were systematically searched for observational studies on patients with VNSSTIs. The primary outcome was all-cause mortality. We did random-effects meta-analysis to obtain estimates for primary outcomes; the estimates are presented as means plus a 95% confidence interval (CI). Data from the selected studies were also extracted and pooled for correlation analyses.

Nineteen studies of 2227 total patients with VNSSTIs were analyzed. More than 95% of the episodes occurred in the subtropical western Pacific and Atlantic coastal regions of the northern hemisphere. While the number of cases and the number of deaths were not correlated with the study period (*r*_s_ = 0.476 and 0.310, *P* = 0.233 and 0.456, respectively), the 5-year mortality rate was significantly negatively correlated with them (*r*_s_ = −0.905, *P* = 0.002). Even so, the pooled estimate of total mortality rates from the random-effects meta-analysis was as high as 37.2% (95% CI: 0.265–0.479).

These data suggest that VNSSTIs are always an important public health problem and will become more critical and urgent because of global warming. Knowing the current distribution of VNSSTIs will help focus education, policy measures, early clinical diagnosis, and appropriate medical and surgical treatment for them.

## INTRODUCTION

Necrotizing skin and soft tissue infections, characterized clinically by fulminant tissue destruction, systemic toxic signs, and high mortality, are broadly classified into 2 categories.^1–3^ Compared with type I (polymicrobial) infections, type II infections are generally monomicrobial and tend to occur on an extremity in younger, healthier patients with a history of known trauma.^3^ Among the pathogens leading to type II infections, group A streptococci (GAS) or other β-hemolytic streptococci are most commonly isolated alone or in combination with other species such as *Staphylococcus aureus*.^4^ Although relatively uncommon globally, some other pathogenic bacteria can also contribute to this highly lethal infectious disease. For example, *Aeromonas hydrophila* (*A. hydrophila*) in fresh water and *Vibrio vulnificus* (*V. vulnificus*) in seawater can cause necrotizing infections due to traumatic injuries. *V. vulnificus* also predisposes to infections in patients with cirrhosis who ingest contaminated raw oysters.^5–7^

*V. vulnificus*, a gram-negative halophilic marine bacillus, is found in water, sediment, plankton, and shellfish such as oysters, clams, and crabs. Many studies^5–7^ report that it undergoes a striking seasonal fluctuation in coastal waters, and that sea surface temperature (SST) is the major factor that promotes bacterial proliferation and controls its persistence and abundance in the aquatic environment.^8,9^ For the past 20 years, the number of reports on *V. vulnificus* necrotizing skin and soft tissue infections (VNSSTIs) has risen. Many scientists believe that higher SSTs promote the spread of *V. vulnificus* in coastal waters and thus contribute to this trend.^9–11^ With global warming, this public health issue will become even more important and urgent. Therefore, we examined the spatiotemporal distribution of the worldwide reported episodes and associated mortality rates of VNSSTIs between 1966 and 2014. The information from this study should be valuable for knowing the current distribution and predicting the spread of VNSSTIs, thereby increasing clinical awareness of them and reducing their mortality rate.

## METHODS

### Search Strategy and Selection Criteria

This systematic review and meta-analysis used the Meta-Analysis of Observational Studies in Epidemiology (MOOSE) criteria.^12^ After being approved by the Institutional Review Board of Chang Gung Memorial Hospital, we searched the PubMed and Cochrane Library databases to identify studies reported on VNSSTIs between January 1966 and December 2014. A search strategy was developed using a combination of free text and controlled vocabulary terms. Search terms included the following: “*Vibrio vulnificus*,∗” “infect,∗” “death,” “mortality,” and “fatality.” We also manually reviewed the references cited in retrieved articles. No language restrictions were placed on the searches or search results. Two independent reviewers (TYY and TSC) assessed all articles considered for inclusion; studies were included only when all reviewers agreed they should be.

### Quality Assessment and Data Extraction

Two reviewers (TYY and TSC) independently assessed the quality of included studies based on the Epidemiological Appraisal Instrument (EAI),^13^ which is a reliable and valid tool for assessing observational studies. Title, author, and journal details were removed to deidentify articles before rating them. Overall agreement for rating article quality and methodology between reviewers was high, and discrepancies were resolved through discussion with the other lead reviewers (KCH, HHW, and MSL). For each study, data were extracted by the first author (KCH) and checked for accuracy by others in regular meetings about study setting, participants (number, mean age, sex, and infection type), and outcome measurements such as mortality.

### Data Analysis and Statistical Methods

Stat 12.1 (StataCorp, College Station, TX) and Open Meta-Analyst were used. Mortality rates were calculated from raw proportions and 95% confidence intervals (CIs) were determined using the Wilson method.^14^ We tested the heterogeneity between combined study results using Cochran Q-test, the degree of inconsistency (*I*^*2*^ values), and then a random-effects model (DerSimonian–Laird method) for the analyses.^15^ Forest plots were generated showing either the mortality rate with corresponding 95% CIs for each study or the overall random-effects pooled estimate. Publication bias was evaluated using methods based on the funnel plot, such as Begg test^16^ and Egger test.^17^ Spearman's rank-based correlation coefficient (*r*_s_) was calculated to study the relationship between the reported number of cases and deaths, the mortality rate, and the study periods. Significance was set at *P* < 0.05 (2-sided).

## RESULTS

**FIGURE 1:**
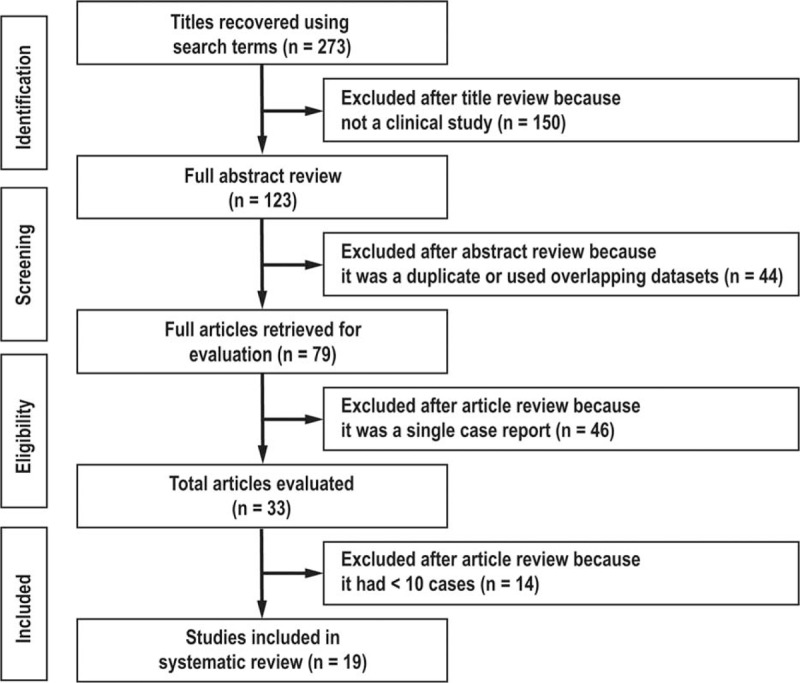
Flow chart of study selection process.

**FIGURE 2:**
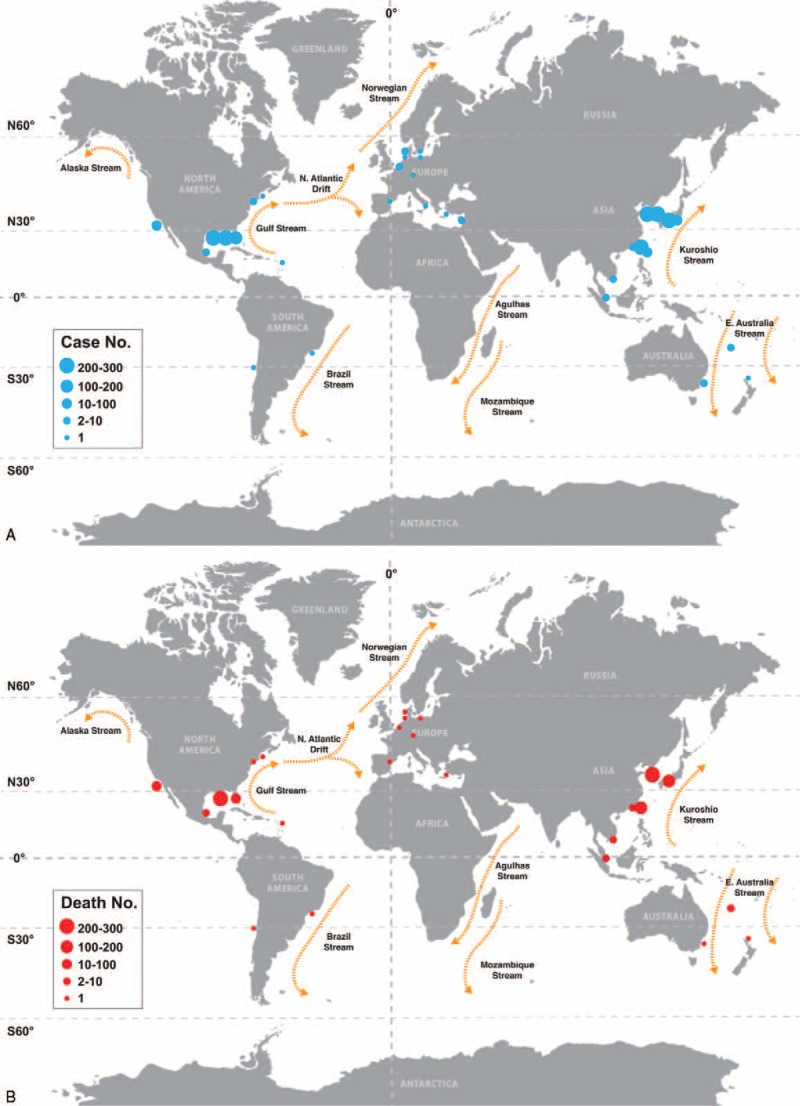
Spatial distribution of the reported (A) total and (B) fatal episodes of *Vibrio vulnificus* necrotizing skin and soft tissue infections.

Figure 1 is a flowchart of the study selection process and shows the number of articles included in the review stages. We examined the titles and abstracts of 273 articles, 150 of which were removed because they were not clinical studies, and 44 because they were duplicates or they shared overlapping datasets. The 79 articles retrieved for evaluation included 46 single-case reports, 14 case series with samples <10, and 19 case series with samples ≥10. The data from these articles were extracted to illustrate the spatial distribution of reported episodes and associated mortality of VNSSTIs in the world between 1966 and 2014. More than 95% of the episodes occurred in the subtropical western Pacific and Atlantic coastal regions of the northern hemisphere (Figure 2).

**FIGURE 3:**
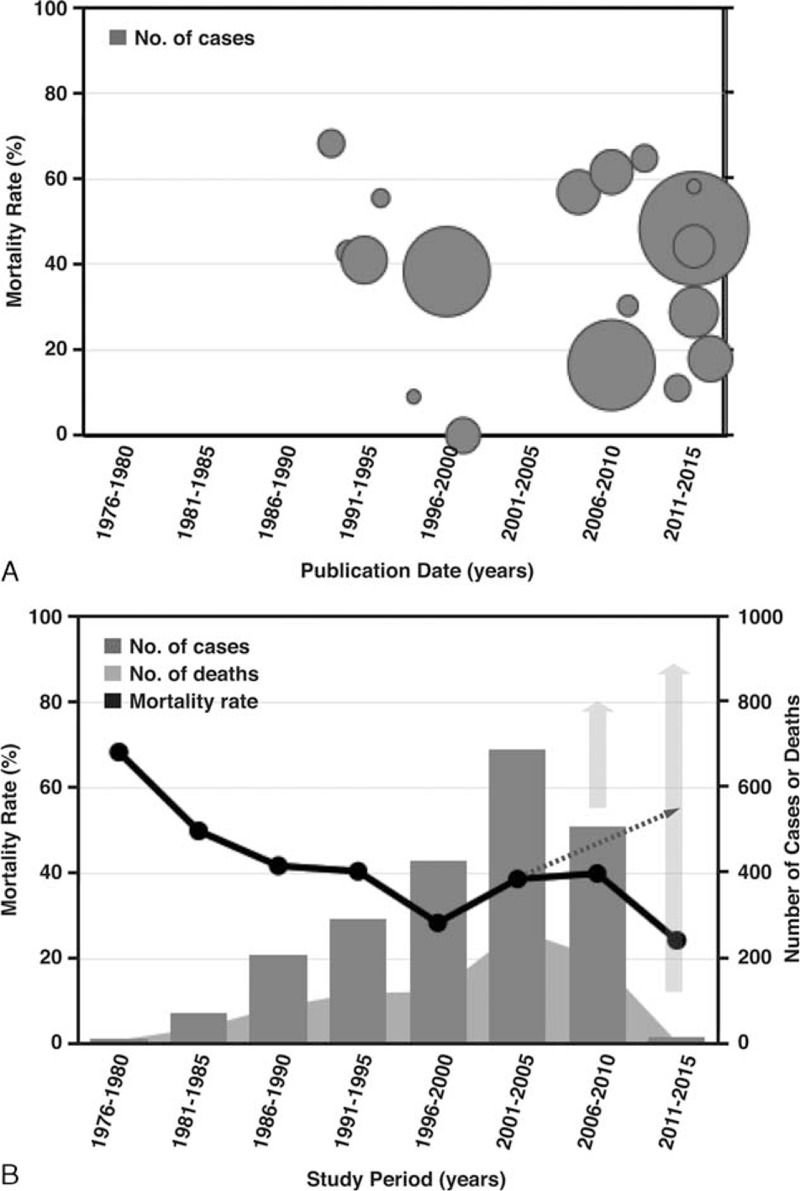
Temporal distribution of the reported episodes of *Vibrio vulnificus* necrotizing skin and soft tissue infections according to (A) publication date and (B) study period. Note: the size of the circles in (A) indicates the reported number of cases in different studies. In (B), the solid gray arrows represent the recruited case numbers from the expected studies in the future and the dashed arrow the projection of mortality based on the actual recorded mortality from 1996 to 2005.

Nineteen articles with samples ≥10 were considered eligible for inclusion in the meta-analysis on mortality of VNSSTIs (Table 1).^18–36^ Regardless of their publication dates and sample sizes, mortality rates varied widely: range = 0–68.4% (Figure 3A). It is interesting that the spots in Figure 3A distributed along a V-shape graph with the vertex located at the lowest 5-year mortality rate in year 2000. After the data had been pooled over time, the number of reported cases and deaths had risen continuously over the past 4 decades (Figure 3B). The calculated 5-year mortality rate had declined by nearly 30%, from 68.4% in 1976 to 1980 to 39.9% in 2006 to 2010. However, it seemed like fit with a parabola distribution that opens upward. The *y*-coordinate of the vertex is around 30% of 5-year mortality rate in years 1996 to 2000. According to the current trends of reported episode growth, the 5-year mortality rate might approach 50% in the future.

**TABLE 1:**
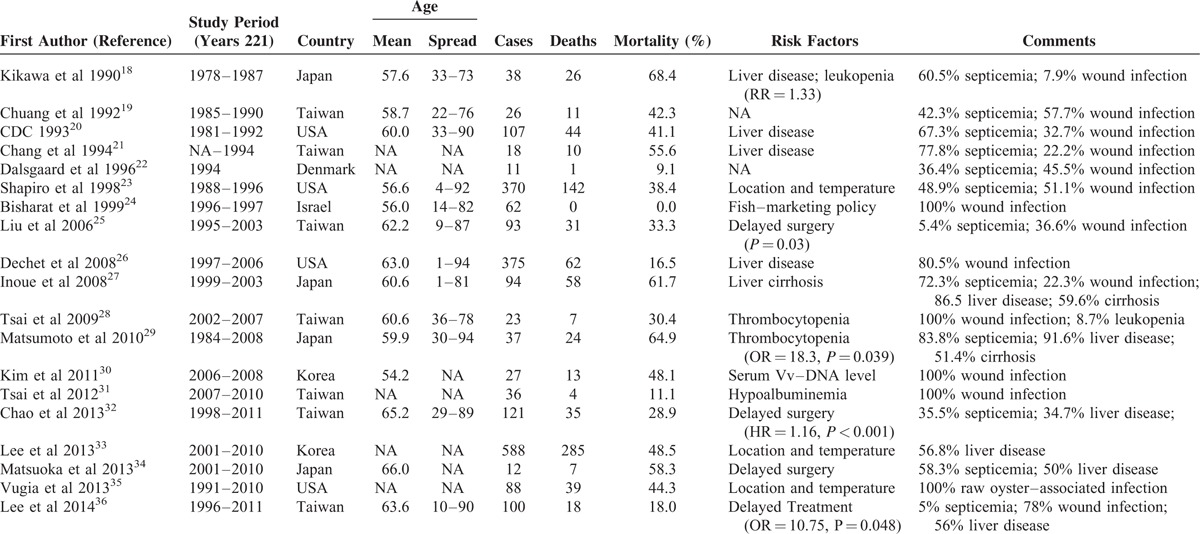
Included Studies of Vibrio Vulnificus Necrotizing Skin and Soft Tissue Infections

**FIGURE 4:**
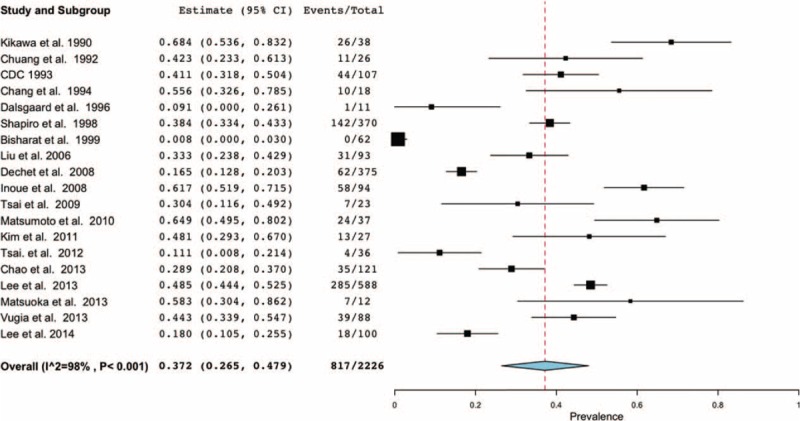
Forest plot of meta-analysis for mortality in patients with *Vibrio vulnificus* necrotizing skin and soft tissue infections. The width of the horizontal line represents the 95% CI of individual studies. The vertical dotted line represents the overall expected mortality. The combined estimate of mortality was 37.2% (95% CI: 26.5–47.9%, *I*^2^ = 98%).

The pooled estimate of mortality rates from the random-effects meta-analysis was 37.2% (95% CI: 0.265–0.479) (Figure 4). The number of reported deaths was strongly positively correlated with the corresponding number of reported cases (*r*_s_ = 0.976, *P* < 0.001). There was no association between the calculated 5-year mortality rate and the number of reported cases (*r*_s_ = −0.429; *P* = 0.289) (Table 2). Regarding the time series patterns, our data showed that the 5-year mortality rate had a strong and significantly negative association with the study period (*r*_s_ = −0.905; *P* = 0.002), but neither the number of cases (*r*_s_ = 0.476; *P* = 0.233) nor the number of deaths did (*r*_s_ = 0.310; *P* = 0.456) (Table 3). Additionally, the funnel plot does not suggest significant publication bias: Begg's test statistic, *P* = 0.972; Egger's test statistic, *P* = 0.106.

**TABLE 2:**
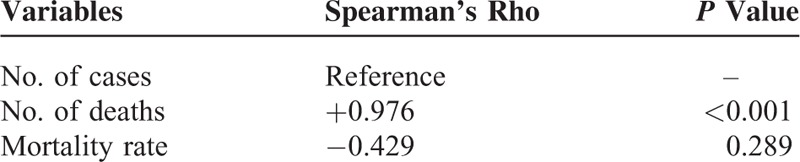
Association Between Number of Reported Cases and That of Reported Deaths/Mortality Rate

**TABLE 3:**
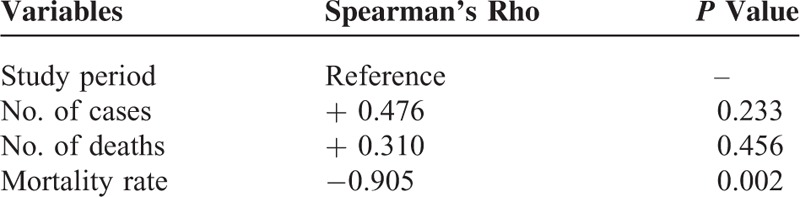
Association Between Study Period and Number of Cases/Deaths/Mortality Rate

## DISCUSSION

We found that more than 95% of the reported episodes of VNSSTIs occurred in the subtropical western Pacific and Atlantic coastal regions of the northern hemisphere, such as Taiwan,^19,21,25,28,31,32,36^ South Korea,^30,33^ Japan,^18,27,29,34^ or the Gulf of Mexico of the United States.^20,23,26,35^ The common average weather feature of these areas is a subtropical monsoon climate pattern, which is rarely seen in the southern hemisphere. From the pooled dataset, we also found, using the random-effects meta-analysis, that there were significantly more reported cases and deaths during the past 4 decades, with an estimated total mortality rate of 37.2%. Although increasing clinical awareness, advancing antibiotics, and improving infection control practices were successful in reducing the mortality rate of VNSSTIs from 1976 to 2000, a rebound or increase in the 5-year mortality rate occurred after it reached its lowest point, that is, around 30% in years 1996 to 2000. Forest plot also showed a similar trend. According to the current trends of reported episode growth, the 5-year mortality rate might approach 50% in the future. These findings highlight that VNSSTIs are always an important public health problem, particularly for people who live in temperate coastal regions.

This public health issue will undoubtedly become even more important and urgent because of global climate change. A study^10^ of the 1996 outbreak of VNSSTIs^24,37^ in Israeli fish market workers found that a high SST because of global warming might have affected the marine ecology of the study area and caused the emergence of the disease. Another study^11^ provided evidence that the pathogenic strain of *V. vulnificus* serovar A, originally isolated in a Spanish eel farm in 2000 and occurring in Denmark 4 years later, had already spread northward into European anguilliculture. The world map of globally warm ocean currents (Figure 2) may provide a clue for understanding the direction and route of the disease spread. The high incidence of VNSSTIs in the subtropical western Pacific coastal region is attributable to the warm SST associated with the Kuroshio Stream, and in the subtropical Atlantic coastal region is attributable to the warm SST associated with the Gulf Stream.^38^ Because of global warming, the higher SST associated with the North Atlantic Drift Current, an extension of the Gulf Stream, greatly influences the distribution, migration, and invasion of *V. vulnificus*, and thus raises the incidence of VNSSTIs in the coastal regions of Continental Europe.^10,11^ We, therefore, recommend that monitoring the nearshore SST because it can provide insights into what ecoregions and coastal residents will be at the greatest risk from this aspect of climate change.^38^

Many studies^30,39–45^ have suggested that the effects of ambient temperature on the pathogenicity of *V. vulnificus* are critical. For example, the abundance and virulence of bacteria in coastal waters and shellfish stock has been strongly linked to water temperature. A field survey study,^39^ done in the Northern Gulf and Atlantic Coast sites from 1994 to 1995, reported that the bacteria density in oysters had slowly increased between 10 and 18°C, more rapidly increased between 18 and 26°C, and then stopped increasing above 26°C. Watanabe et al^40^ found that the virulent, but not the avirulent, strains proliferated sufficiently in tryptone yeast extract broth containing 0.9% NaCl when cultivated at 37°C, which mimicked the conditions in human plasma. Concomitantly, these rapidly multiplying virulent pathogens produced more toxic proteases than did others, which were thought to be the major pathogenic factors of *V. vulnificus*.^41–45^ Moreover, Kim et al,^30^ in a study on the association between the *V. vulnificus* DNA load and mortality in patients with VNSSTIs, said that the DNA level was significantly higher in nonsurvivors than in survivors. All these findings provide an explanation for the high total mortality rate associated with VNSSTIs despite our increasing clinical awareness, and advances in antibiotic and infection control practices over the past 20 years. Education and healthcare policies are, therefore, needed to combat this emerging infectious disease.

Cooling shellfish immediately after they have been harvested and carefully storing them between 0 and 4°C might reduce their number of pathogenic *V. vulnificus* and its threat to public health.^46^ The 1996 VNSSTIs outbreak in Israel^10,24,37^ provides a good example that such changes in fish-marketing policy do affect disease outbreaks. Although cold storage does reduce the risk of VNSSTIs, it cannot be relied upon to totally eliminate the organism. Studies^47–49^ have found that bacterial cultures held at 4°C and below undergo a time-dependent reduction in the number of recoverable cells; however, the time required for the bacteria to reach undetectable levels might exceed their usual storage life. Moreover, *V. vulnificus* can enter a form of dormancy known as the viable but nonculturable (VBNC) state,^50^ which permits their resurrection when the temperature rises sufficiently. Because of these limitations, we conclude that clinical awareness is paramount for diagnosing and treating these highly lethal and disabling VNSSTIs, particularly in immunocompromised patients.

In conclusion, VNSSTIs are always an important public health problem, and they are likely to become more critical and urgent because of the rising SST associated with global warming. Knowing the current spatiotemporal distribution of these emerging infectious diseases will help focus education, policy measures, early clinical diagnosis, and appropriate medical and surgical treatment for them. Although careful cold storage can greatly reduce their threat, clinical awareness is crucial for diagnosing and treating patients with suspected VNSSTIs, particularly immunocompromised patients.
